# Homozygosity in the *APOE* 3 Polymorphism Is Associated With Less Depression and Higher Serum Low-Density Lipoprotein in Chinese Elderly Schizophrenics

**DOI:** 10.3389/fendo.2020.00642

**Published:** 2020-10-15

**Authors:** Wei Li, Chunxia Ban, Ling Yue, Lin Sun, Xia Li, Shifu Xiao

**Affiliations:** ^1^Department of Geriatric Psychiatry, Shanghai Mental Health Center, Shanghai Jiao Tong University School of Medicine, Shanghai, China; ^2^Alzheimer's Disease and Related Disorders Center, Shanghai Jiao Tong University, Shanghai, China; ^3^General Psychiatry, Jiading District Mental Health Center, Shanghai, China

**Keywords:** aging, APOE E3, depressive symptom, Chinese, schizophrenia

## Abstract

**Background:** Depressive symptoms are common comorbidities in schizophrenia. However, the effect of APOE E3 on depressive symptoms has never been investigated in an aging Chinese population with schizophrenia. This cross-sectional study aimed to investigate the effects of APOE E3 on blood lipid metabolism and depressive symptoms in elderly schizophrenics in China.

**Methods:** Three Hundred and one elderly schizophrenics (161 males, age ranges from 60 to 92 years, with an average age of 67.31 ± 6.667) were included in the study. Depressive symptoms were assessed using the Geriatric Depression Scale (GDS). APOE gene polymorphism was determined by polymerase chain reaction (PCR). We assessed the correlations of GDS and serum low-density lipoprotein (LDL) with APOE genotypes.

**Results:** The concentration of LDL in the Homozygous APOE E3 group was significantly higher than that in the non-homozygous APOE E3 group, while the scores of GDS of the Homozygous APOE E3 group were lower than that in the non-homozygous APOE E3 group. Using partial correlation analysis and controlling age, gender, duration of disease, and hyperlipidemia, we found that the scores of GDS were significantly correlated with LDL (*r* = −0.194, *p* = 0.016).

**Conclusions:** APOE E3 is associated with less depressive symptoms and higher serum LDL in Chinese elderly patients with schizophrenia, and there is a negative correlation between depressive symptoms and LDL.

## Introduction

Schizophrenia is associated with an increased prevalence of depressive symptoms. According to previous studies, ~59% of schizophrenia patients suffer from either minor or major depression (1, 2). Depressive symptoms are commonly seen in all stages of schizophrenia, particularly in the acute phase (3). They have been found to correlate with positive symptoms, negative symptoms, and general psychopathology in patients with schizophrenia (4). The accumulated evidence from previous studies suggests that the presence of depressive symptoms in patients with schizophrenia has been associated with worse overall outcomes, greater comorbidity, work impairment, poorer quality of life, deterioration of psychosocial functioning, greater risk of relapse, and increased risk of suicide (5, 6). It is therefore extremely important to understand the prevalence and influencing factors of depression in schizophrenics and to develop effective interventions.

Various physiological factors increase the risk of depressive symptoms or depression, including lipid metabolism and genetic variations related to lipids (7). A potential genetic variant that affects lipid metabolism symptoms is the Apolipoprotein E (APOE) gene. APOE is critical in the modulation of phospholipid and cholesterol transport between cells, and it is also believed to play a significant role in neuronal growth and repair (8). Human APOE is a polymorphic protein, APOE E2, E3, and E4, which are encoded by three alleles, ϵ2, ϵ3, and ϵ4, respectively (9). Meta-analyses of genetic studies reported an association of APOE allele ϵ2 with major depressive disorder (10). Skoog et al. (11) proved that the presence of APOE allele ϵ4 predicted future depression and might be an identifier for aging people who are at high risk of clinically significant depression.

The APOE E3 allele is the most frequent allele in all human groups (12) and has been reported to have protection against cardiovascular diseases by maintaining lipid homeostasis (13). However, the effect of APOE E3 on depressive symptoms in schizophrenia has never been investigated. In response, we conducted a cross-sectional study to investigate the effects of homozygosity in the APOE 3 polymorphism on lipid metabolism and depressive symptoms in elderly Chinese schizophrenics.

## Materials and Methods

### Participants

This cross-sectional study was conducted between July 1, 2015, and December 31, 2015, and included 301 hospitalized elderly schizophrenics (age ranges from 60 to 92 years, with an average age of 67.31 ± 6.667; among them, 161 were males, accounting for 53.5%), who were recruited from three mental health centers (including the Shanghai Mental Health Center, the Mental Health Center of Fengxian District in Shanghai, and the Mental Health Center of Jiading District in Shanghai). The method of sampling has been described in our previous studies (14). The inclusion criteria were as follows: (1) aged 60 or more; (2) diagnosed with schizophrenia, which was diagnosed by a senior psychiatrist according to the International Classification of Diseases 10 diagnostic standard; (3) without major medical abnormalities, including unstable, acute, or life-threatening medical illness and central nervous system diseases; (4) was able to cooperate and complete relevant inspections. Subjects with a history of major medical abnormalities (e.g., cancer and infection) and those who chose not to take part were excluded. Through face-to-face interviews, we obtained general demographic data (for example, age, education, gender, BMI, duration of disease), daily living habits (smoking, drinking, drinking tea, physical exercise, and hobbies), disease history (hypertension, diabetes, and hyperlipidemia) and currently prescribed medicines (including clozapine, olanzapine, quetiapine, risperidone, and aripiprazole).

This study was approved by the Research Ethical Committee of the affiliated mental health center of the Shanghai Jiaotong University, School of Medicine. Written informed consent was obtained from all participants before the study. All research processes were conducted according to the principles of the Declaration of Helsinki.

### Neuropsychological Assessment

#### Depression Evaluation

Depression is a condition characterized by a depressed mood or loss of pleasure or interest in nearly all activities almost every day for at least 2 weeks (15). The presence of depressive symptoms was determined using the Geriatric Depression Scale (GDS) (16). The GDS consists of 30 items (hereafter referred to as the GDS-30), and participants were asked to answer “yes” or “no” to these items based on how they felt over the past week, and those with a score higher than 10 points were considered to have depression (17).

### Cognitive Assessment

The Montreal Cognitive Assessment (MoCA) was used to evaluate the cognitive function of these subjects. MoCA is a widely used 10-min cognitive screening test for the detection of mild cognitive impairment (MCI), with high sensitivity (90%) and specificity (87%) (18). It has been proven to be effective in detecting cognitive impairment in schizophrenics (19).

### Symptom Assessment

The Positive and Negative Syndrome Scale (PANSS) was utilized to assess the symptoms and severity of schizophrenia, as it is a reliable and valid instrument that has served the scientific research community for decades (20). The PANSS includes four scales measuring positive and negative syndromes, aggressiveness, as well as general severity of illness (21). Several subsequent studies have shown that it has strong psychometric properties in terms of reliability, validity, and sensitivity (22).

### Genotyping of APOE and Biochemical Detection of Blood Lipids

The genomic DNA was removed from peripheral blood (Morning fasting whole blood) by using a Blood Genomic DNA Extraction Kit (spin column, Tiangen Biochemical Science and Technology Co., Ltd., Beijing, China). The APOE genotype was determined by multiplex amplification refractory mutation system polymerase chain reaction (PCR). The multiplex PCR reactions based on the two SNP cores of the APOE gene were measured using the rs7412 and rs429358 design primers as follows: P1:5-′GCCTACAAATCGGAACTGGACAGCTCCTCGGTGCTCTG-3′ and P2:5 ′-TAAGCGGCTCCTCCGCGATGCCCCGGCCTGGTACACTG-3′. We verified the primers on Nucleotide-nucleotide BLAST: (https://ncbiinsights.ncbi.nlm.nih.gov/tag/nucleotide-blast).

According to the methods previously described (23), the 301 subjects were divided into two groups, the Homozygous APOE E3 group (ε3/ε3, *n* = 205) and the non-homozygous APOE E3 group (ε2/ε2, ε2/ε3, ε3/ε4, and ε4/ε4, *n* = 96) group. Tables 1 and 2 list the information about gene distribution in detail. The values of serum triglyceride, cholesterol, fasting blood glucose, low-density lipoprotein, and high-density lipoprotein were obtained by using the hexokinase method on an auto-analyzer (Dimension Xpand plus).

**Table 1:** Allele frequencies and prevalence of APOE among Chinese elderly with schizophrenia.

**APOE**	**Male (*n* = 161)**	**Female (*n* = 140)**	**Combined (*n* = 301)**
E2 (ϵ2/ϵ2, ϵ2/ϵ3)	23 (14.3%)	17 (12.1%)	40 (13.3%)
E3(ϵ3/ϵ3)	109 (67.7%)	96 (68.6%)	205 (68.1%)
E4(ϵ2/ϵ4, ϵ3/ϵ4, ϵ4/ϵ4)	29 (18.0%)	27 (19.3%)	56 (18.6%)
ϵ2/ϵ2	1 (0.6%)	1 (0.7%)	2 (0.7%)
ϵ2/ϵ3	22 (13.7%)	16 (11.4%)	38 (12.6%)
ϵ2/ϵ4	2 (1.2%)	4 (2.9%)	6 (2.0%)
ϵ3/ϵ3	109 (67.7%)	96 (68.6%)	205 (68.1%)
ϵ3/ϵ4	23 (14.3%)	20 (14.3%)	43 (14.3%)
ϵ4/ϵ4	4 (2.5%)	3 (2.1%)	7 (2.3%)

**Table 2:** General demographic data of the Chinese elderly with schizophrenia based on APOE E3.

**Variables**	**Homozygous APOE E3 (*N* = 205)**	**Non-homozygous APOE E3 (*N* = 96)**	**F or X^**2**^**	***P***
Age, y	67.10 ± 6.542	67.73 ± 6.888	−0.768	0.443
Education, y	8.21 ± 3.652	7.63 ± 3.807	1.272	0.204
Duration of disease, y	35.45 ± 13.241	37.71 ± 13.066	−1.374	0.170
BMI, kg/m^2^	23.95 ± 4.217	23.56 ± 3.989	0.749	0.455
Fasting blood glucose, mmol/L	5.50 ± 1.491	5.47 ± 1.264	0.177	0.859
Triglyceride, mmol/L	1.38 ± 0.836	1.39 ± 0.808	0.076	0.927
High density lipoprotein, mmol/L	1.29 ± 0.406	1.31 ± 0.412	−0.055	0.956
Low density lipoprotein, mmol/L	2.83 ± 0.752	2.62 ± 0.858	2.140	0.033^*^
Male, n (%)	109 (53.2)	52 (54.2)	0.026	0.902
Hypertension, n (%)	78 (38.0)	33 (34.4)	0.379	0.609
Diabetes, n (%)	52 (25.4)	26 (27.1)	0.100	0.779
Hyperlipidemia, n (%)	72 (35.1)	48 (50.0)	6.037	0.016^*^
Smoker, n (%)	69 (33.7)	28 (29.2)	0.604	0.509
Drinker, n (%)	24 (11.7)	9 (9.4)	0.364	0.693
Tea drinker, n (%)	44 (21.5)	22 (22.9)	0.081	0.767
Physical exercise, n (%)	66 (32.2)	29 (30.2)	0.119	0.791
Hobby, n (%)	78 (38.0)	31 (32.3)	0.938	0.369
Clozapine, n (%)	33 (16.1)	15 (15.6)	0.011	1.000
Olanzapine, n (%)	54 (26.3)	28 (29.2)	0.263	0.677
Quetiapine, n (%)	26 (12.7)	16 (16.7)	0.864	0.375
Risperidone, n (%)	61 (29.8)	26 (27.1)	0.227	0.684
Aripiprazole, n (%)	38 (18.5)	17 (17.7)	0.030	1.000
MoCA	14.23 ± 6.740	12.99 ± 7.203	1.335	0.183
GDS	9.46 ± 5.927	11.71 ± 5.514	−2.765	0.006^*^
PANSS	63.95 ± 21.100	65.70 ± 22.823	−0.603	0.547

* *p < 0.05*.

### Statistical Analysis

Continuous variables were expressed as mean ± SD and categorical variables were expressed as frequencies (%). A single sample Kolmogorov-Smirnov test was used to test whether the data conformed to the normal distribution and an independent sample *t*-test was used to compare the data of normal distribution between the Homozygous APOE E3, and the non-homozygous APOE E3 group. The Mann-Whitney U test was used to compare the data of non-normal distribution, while the Chi-square test was used to categorical variables between the two groups. The partial correlation analysis was then used to explore the association between neuropsychological tests and blood lipids (age, education, duration of disease, and hyperlipidemia were controlled). All statistical analyses were performed using SPSS 22.0 (IBM Corporation, Armonk, NY, USA), and two-tailed tests were utilized at a significance level of *P* < 0.05.

## Results

Table 1 presents the results of the allele and genotype frequencies. Frequencies of APOE E3 were greatest (68.1%). Table 2 displays the characteristic of subjects with different APOE genotypes. By using a single sample Kolmogorov-Smirnov test, we found that BMI (*p* > 0.05) was in the normal distribution, while age, education, duration of disease, fasting blood sugar, triglyceride, high- density lipoprotein, low-density lipoprotein, and scores of MoCA, GDS, and PANSS (*p* < 0.05) were in non-normal distribution.

**Figure 1:**
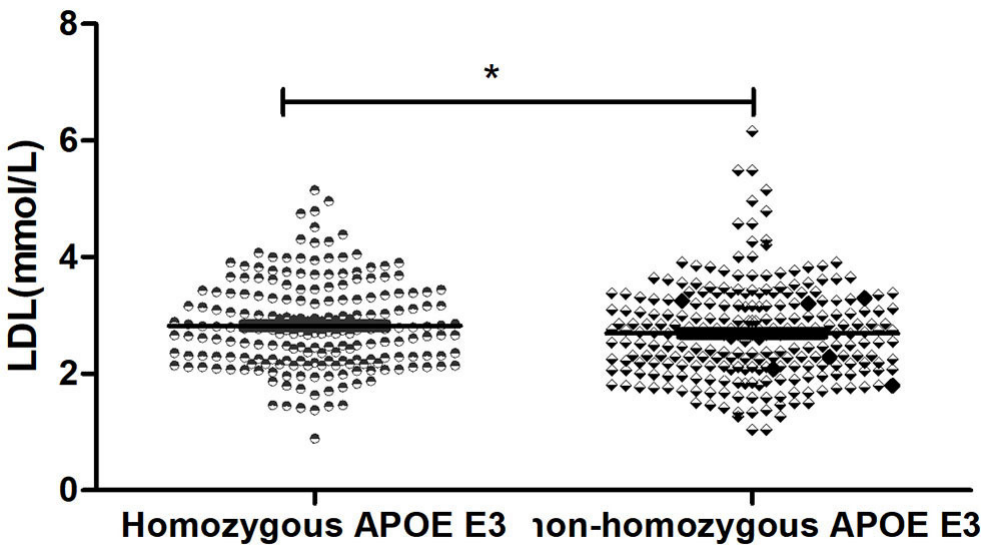
Comparsion of LDL between two groups. *means *p* < 0.05.

**Figure 2:**
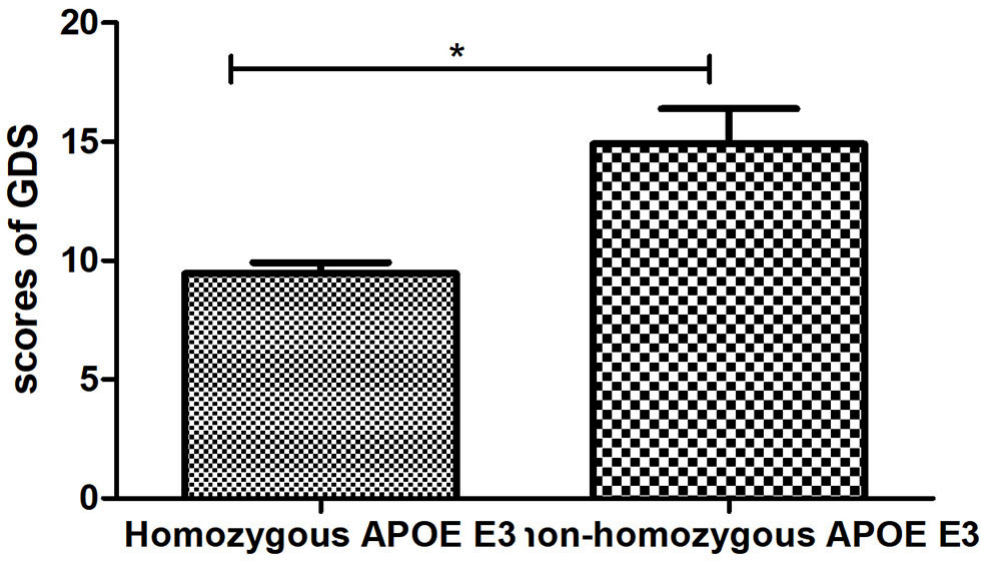
Comparsion of GDS between two groups. *means *p* < 0.05.

By using the Independent sample *t*-test (Normal distribution), Mann-Whitney U test (Non-normal distribution), and Chi-square test (Categorical variable), we found that there were statistical differences between the two groups in terms of low density lipoprotein (*p* = 0.033), the score of GDS (*p* = 0.006), and hyperlipidemia (*p* = 0.016). There was no significant difference (*p* > 0.05) in age, education, duration of disease, BMI, fasting blood sugar, triglyceride, high-density lipoprotein, gender, hypertension, diabetes, smoker, drinker, tea drinker, physical exercise, hobby, clozapine, olanzapine, quetiapine, risperidone, aripiprazole, and scores of MoCA and PANSS between the two groups (Table 2). The concentration of LDL in the Homozygous APOE E3 group was significantly higher than the non-homozygous APOE E3 group, while the scores of GDS in the Homozygous APOE E3 group were lower than that in the non-homozygous APOE E3 group. Table 2, Figures 1, 2 show the results. By using partial correlation analysis and controlling age, gender, duration of disease, and hyperlipidemia, we found that the scores of GDS were significantly correlated with low-density lipoprotein (*r* = −0.194, *p* = 0.016).

## Discussion

To the best of our knowledge, this is the first study to explore the effect of homozygosity in the APOE 3 polymorphism on depressive symptoms in a Chinese elderly population with schizophrenia. The study revealed several interesting findings: (1) that the APOE E3 genotype was associated with less depressive symptoms in elderly patients with schizophrenia; (2) the APOE E3 genotype was associated with higher serum low-density lipoprotein; and, (3) that there was a negative correlation between low-density lipoprotein and depression score.

The present study recruited 301 hospitalized elderly schizophrenics. All participants completed a neuropsychological assessment (MoCA, GDS, and PANSS), APOE gene polymorphism test, and blood lipid test. Using the Mann-Whitney U test, we found that APOE E3 was associated with lower depressive symptoms and higher concentrations of LDL. By using partial correlation analysis and controlling age, gender, duration of disease, and hyperlipidemia, we proved that scores of GDS were significantly correlated with low density lipoprotein (*r* = −0.194, *p* = 0.016).

To verify whether the above conclusions still exist in a non-schizophrenic elderly population, we recruited 154 participants as controls (age ranges from 60 to 78 years, with an average age of 68.54 ± 6.632). Among them, 72 were male, accounting for 46.8%, and there was no statistical difference in the above indexes between the schizophrenia group and control group. After controlling for age and gender, there was no statistical difference in the concentration of LDL between the Homozygous APOE E3 group and the non-homozygous APOE E3 group. However, the scores of GDS in the Homozygous APOE E3 group were still lower than those in the non-homozygous APOE E3 group. Table 3 presents these results.

**Table 3:** Characteristics of Chinese normal elderly subjects with different APOE groups.

**Characteristics**	**Homozygous APOE E3 (*n* = 97)**	**Non-homozygous APOE E3 (*n* = 57)**	**t or X^**2**^**	***p***
Age, y	68.30 ± 6.515	68.95 ± 6.867	−0.585	0.560
Low density lipoprotein, mmol/L	2.874 ± 0.870	2.930 ± 0.809	−0.401	0.689
Male, n (%)	45 (46.4)	27 (47.4)	0.014	1.000
GDS	4.769 ± 3.947	6.887 ± 4.718	−2.887	0.005^*^

* *p < 0.05*.

The relationship between APOE gene polymorphism and schizophrenia is very complicated. Gibbons et al. (24) have pointed out that alterations in APOE expression might lead to either the risk of developing schizophrenia or clinical manifestations, as APOE was important in CNS functions such as modulating cell signaling, protein phosphorylation, intraneuronal calcium, and storage-induced synaptic sprouting. However, other related conclusions are not consistent. For example, a French association study and meta-analysis suggested that there was no major role for APOE gene variants in schizophrenia as a whole (25). However, another study supported that the APOE epsilon 4/epsilon 4 genotypes might be associated with early-onset schizophrenia, while the APOE epsilon 3 allele might function protectively in later onset in this disease (26). Therefore, a large sample of longitudinal studies is needed to further examine the association between APOE gene polymorphism and schizophrenia.

It is well-known that lipid metabolism could be affected by the frequency of APOE (27). In our study, we found that APOE E3 was associated with higher LDL in Chinese aging patients with schizophrenia and there was a negative correlation between LDL and depression score. A systematic review and meta-analysis (28) showed that patients with depression tend to have lower LDL (Mean difference = −4.29, 95% *CI* = −8.19, −0.40, *p* = 0.03). Beasley et al. (29) found that cholesterol levels were 13% lower in major depressive disorder (*p* = 0.018) and 10% lower in bipolar disorder (*p* = 0.052) compared with controls, while there was no significant difference (*p* > 0.05) between schizophrenia and controls. Another study by Olsson et al. (30) also indicated that low LDL might increase the risk of depression and the mechanism might involve immunity, inflammation, and brain dysfunction. However, a previous study suggested that stroke patients with the APOE genotype ϵ3/ϵ3 had more symptoms of depression compared to the other genotypes (13). Another study showed that there was no significant association between depression and APOE genotype (31). Our findings are partially consistent, and different human populations and different disease types might be responsible for these differences.

Several mechanisms might be used to explain why the APOE E3 genotype helps prevent depression. First, APOE E3 can inhibit amyloid accumulation and increase LDL receptor levels. Second, APOE E3 also plays a crucial role in cholesterol efflux and reverse cholesterol transport, and bears anti-inflammatory and anti-oxidant properties (32). Third, E3 ubiquitin ligase IDOL determines the level of ApoER2 protein in the synapse in response to neuronal activation and regulates the morphogenesis and plasticity of dendritic spines. Furthermore, APOE E3 contributes toward maintaining the integrity of endothelial function and the blood-brain barrier (BBB) at the neurovascular junction (33).

There are some limitations to our research. First, this is a cross-sectional study, unable to establish the causal relationship between APOE E3, LDL, and depressive symptoms. Second, the relatively small sample size reduces the reliability of the study. Third, these subjects have been taking antipsychotics, which could have influenced the research conclusion.

## Conclusions

The APOE E3 genotype is associated with less depressive symptoms and higher serum low-density lipoprotein in Chinese elderly patients with schizophrenia. There is a negative correlation between depressive symptoms and low-density lipoprotein. Therefore, it may be possible to improve depressive symptoms by increasing blood lipid concentration in patients with schizophrenia, but this conclusion needs to be verified by a large sample of longitudinal research.

## Data Availability Statement

The datasets analyzed in this article are not publicly available, because the database is less than ten years old, and it needs to be authorized by the data owner to be public. Requests to access the datasets should be directed to ja_1023@hotmail.com.

## Ethics Statement

The studies involving human participants were reviewed and approved by Research Ethical Committee of the affiliated mental health center of Shanghai jiaotong university school of medicine. The patients/participants provided their written informed consent to participate in this study. Written informed consent was obtained from the individual(s) for the publication of any potentially identifiable images or data included in this article.

## Author Contributions

WL and LS contributed to the study concept and design. LY and CB acquired the data. XL and SX analyzed the data and drafted the manuscript. All authors read and approved the final manuscript.

## Conflict of Interest

The authors declare that the research was conducted in the absence of any commercial or financial relationships that could be construed as a potential conflict of interest.
